# International Network to Promote Household Water Treatment and Safe Storage

**DOI:** 10.3201/eid1006.040243

**Published:** 2004-06

**Authors:** Thomas F. Clasen, Eric D. Mintz

**Affiliations:** * London School of Hygiene and Tropical Medicine, London, United Kingdom;; † Centers for Disease Control and Prevention, Atlanta, Georgia, USA

**Keywords:** waterborne disease, chlorination, filtration, diarrhea prevention, news and notes

On February 25, 2003, more than 30 representatives from United Nations agencies, international nongovernmental organizations, research institutions, professional associations, and private companies met in Geneva, Switzerland, to establish the International Network to Promote Household Water Treatment and Safe Storage, sponsored by the World Health Organization (WHO) (Figure). The group has since convened at the Kyoto World Water Forum, Kyoto, Japan, in Washington, D.C., and in Cape Town, South Africa. At the next plenary meeting, in Nairobi, Kenya, on June 14–15, 2004, participants will consider a 5-year strategic plan to reduce waterborne disease through specific actions in research, advocacy, communication, and implementation.

**Figure:**
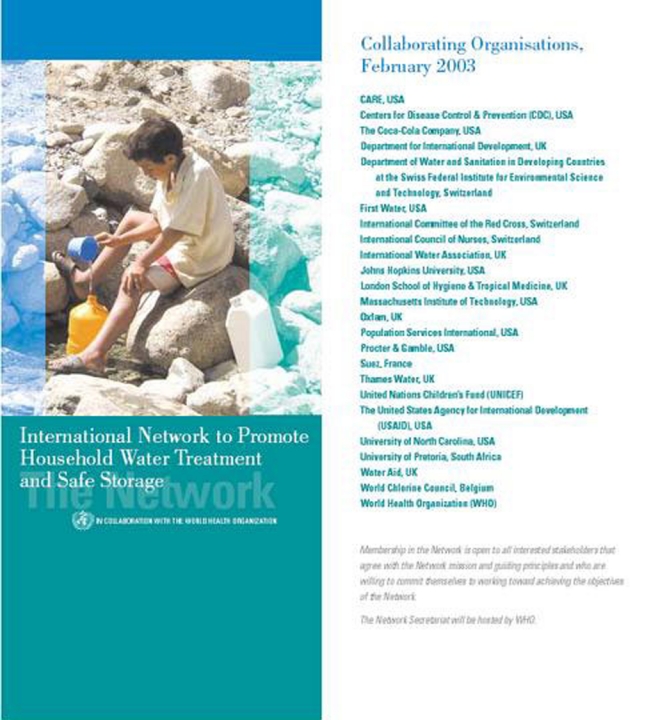
Cover and inside page of brochure, The Network.

This first phase has seen progress in the organization and expansion of the network's participant base and real work in the field, building on the evidence that household water management can significantly contribute to meeting the Millennium Development Goals for child survival and water security. This fieldwork has reaffirmed the conclusion of a WHO-sponsored review: simple, low-cost interventions for home water treatment and storage lead to dramatic improvements in drinking water quality and reductions in diarrheal disease (*1*).

The Safe Water System, developed by the Centers for Disease Control and Prevention (CDC), the Pan American Health Organization (PAHO), and WHO, combines point-of-use water disinfection with locally produced sodium hypochlorite, safe storage in narrow-mouth containers, and community education and has consistently been effective in preventing diarrhea (*2**,**3*). In recently published trials, the Safe Water System reduced diarrhea by 24% in Bangladesh (*4*) and 25% in Guatemala (*5*). In a 2003 study, the Safe Water System reduced diarrhea by 30% among persons with HIV infection in rural Uganda (*6*).

In 2003, accounts of field trials of a household-based flocculant-disinfectant for water treatment were published for the first time. Developed by the Procter & Gamble Company and CDC, the intervention combines a chemical flocculant with a timed-release hypochlorite disinfectant. Through precipitation, coagulation, and flocculation, the combined product physically removes a broad range of microbial pathogens and chemicals, including arsenic, and concurrently inactivates remaining microbes with free chlorine (*7**,**8*). In a randomized, controlled trial in Guatemala, use of the product reduced the incidence of diarrhea among intervention households by 24%, or 29% when the treated water was stored in a vessel designed specifically for safe storage (*5*).

In 2003, considerable progress was made in evaluating the impact of household-based filtration. In a large field trial, Rita Colwell and colleagues showed that simple filters made from sari cloth or nylon, combined with appropriate education, reduced cholera by 48% compared to controls (*9*). Locally produced slow sand and ceramic filters were evaluated by Massachusetts Institute of Technology postgraduate students (*10**–**12*). In a trial in Bolivia, locally fabricated filters that used imported ceramic candles eliminated all detectable fecal coliform bacteria in household drinking water and reduced levels of diarrhea by 64% (*13*).

In 2004, a systematic review of 57 studies assessed the extent and causes of microbiological contamination of household drinking water between the source and the consumer (*14*). The reviewers concluded that water quality declines substantially after collection and recommended household treatment and safe storage of water. A systematic review of the health impact of improved water quality is under way, driven in part by the burgeoning evidence indicating that substantial health gains result when water is treated in households and protected against recontamination (*15**,**16*).

In Nairobi, network members will review recent progress and plan their next steps for advancing household-based water management. Stakeholders from all organizations are urged to participate in, contribute to, and take full advantage of, this important new movement in the battle against waterborne disease. For more information on the network and to register for the Nairobi meeting, readers are referred to: http://www.cdc.gov/safewater/network.htm
